# Phytoremediation of Heavy Metal-Contaminated Soil Using Drought-Adapted Sweet Sorghum (*Sorghum bicolor* L.) in Arid Regions of Kazakhstan

**DOI:** 10.3390/plants14233627

**Published:** 2025-11-28

**Authors:** Aigerim M. Sagimbayeva, Nasya B. Tomlekova, Galymzhan A. Saparov, Yergali O. Abduraimov, Aslan A. Kerimbayev, Sergazy Sh. Nurabayev, Nurika N. Assanzhanova, Nurlan Zh. Akmyrzayev, Konirsha M. Iskakova, Aiman Sh. Omarova, Bakytzhan B. Anapiyayev

**Affiliations:** 1 Research Institute for Biological Safety Problems, National Holding “QazBioPharm”, Gvardeiskiy 080409, Kazakhstans.nurabayev@biosafety.kz (S.S.N.); n.assanzhanova@biosafety.kz (N.N.A.); n.akmyrzayev@biosafety.kz (N.Z.A.); 2 Agricultural Academy, Maritsa Vegetable Crops Research Institute, 4000 Plovdiv, Bulgaria; nasia.tomlekova@gmail.com; 3 Kazakh Research Institute of Soil Science and Agrochemistry Named After U. U. Uspanov, Almaty 050060, Kazakhstan; 4 Department of Chemical and Biochemical Engineering, Satbayev University, Almaty 050013, Kazakhstan; 5 Institute of Agriculture and Plant Growing, Almalybak 040909, Kazakhstan

**Keywords:** heavy metals, phytoremediation, sweet sorghum, translocation, phytoextraction, phytostabilization, *Sorghum bicolor* L.

## Abstract

Soil contamination with heavy metals is a persistent challenge in the arid regions of Kazakhstan. This study evaluates the phytoremediation potential of sweet sorghum (*Sorghum bicolor* L.), a drought-tolerant crop with a well-developed root system, using a combination of in vitro and analytical approaches. In vitro culture of somatic cells revealed clear genotype-dependent differences in callus induction and morphogenesis, with Hybrid-2 and SAB-3 exhibiting the highest regenerative capacity and thus the greatest suitability for further biotechnological improvement and stress-tolerance selection. Analysis of metal distribution, based on atomic absorption spectroscopy (AAS), demonstrated that *S. bicolor* predominantly retained Pb, Cd, and Co in the root system. Cobalt accumulated to 12.7 ± 1.32 mg/kg under 1 MAC and 16.87 ± 2.78 mg/kg under 2 MAC, accounting for more than half of the metal absorbed by plants. Cadmium showed a similar root-dominant pattern, whereas lead exhibited the lowest mobility and remained almost entirely sequestered in roots, with translocation factors consistently below unity (TF < 1). Overall, these findings confirm the suitability of sweet sorghum as an environmentally sustainable species for the phytostabilization of Pb-, Cd-, and Co-contaminated soils in arid environments and highlight the value of genotype pre-selection under stress conditions for optimizing phytoremediation performance.

## 1. Introduction

Environmental pollutants are toxic substances introduced into ecosystems from both anthropogenic and natural sources. Certain industrial processes—such as synthetic chemical manufacturing, coal processing, and the environmentally problematic practice of waste incineration—pose significant risks to environmental components. These include abiotic elements such as air and water, as well as the soil environment, which represents a complex matrix composed of both biotic and abiotic constituents. Such pollutants also pose major threats to biological communities, including plants, animals, and humans [1,2,3]. Lead, cadmium, and cobalt are chemically stable elements that can remain in soils for very long periods; their environmental impact is determined primarily by soil factors (such as pH, organic matter content, and redox conditions) that control their mobility and bioavailability [4].

Kazakhstan has been experiencing widespread soil contamination with heavy metals as a result of decades of intensive mining, metallurgical, and industrial activities, particularly in the Pavlodar, East Kazakhstan, Karaganda, and Turkestan regions. Elevated concentrations of lead (Pb), cadmium (Cd), and cobalt (Co) frequently exceed both national and international regulatory limits [5,6].

Removing heavy metals from the environment is highly challenging, as these elements are neither biologically nor chemically degradable. Conventional remediation strategies for contaminated soils include ex situ and in situ technologies, such as chemical reduction, electrokinetic remediation, excavation, soil washing, solidification, and vitrification. Traditional “pump-and-treat” or “dig-and-dump” approaches are generally limited to small-scale sites and suffer from significant technical, economic, and ecological constraints [7].

Heavy metals can be highly toxic to living organisms, causing genotoxic, teratogenic, and mutagenic effects once their concentrations rise above the narrow physiological range. Essential trace metals (e.g., Fe, Zn, Cu, Mn, Co) are required for normal metabolism, whereas non-essential metals such as Cd and Pb may induce endocrine and neurological disturbances even at comparatively low exposure levels [8,9,10].

In recent decades, plant-based environmental restoration—particularly phytoremediation—has garnered considerable global interest as a sustainable strategy for decontaminating polluted soils [11,12]. Among emerging remediation techniques, phytoremediation is now widely regarded as one of the most promising approaches in industrially developed regions [13]. Certain plant species naturally thriving on contaminated sites can accumulate substantial amounts of heavy metals in their tissues without exhibiting visible phytotoxic symptoms [14]. This method is cost-effective, environmentally benign, operationally simple, and applicable across diverse and ecologically degraded landscapes [15,16,17,18].

Phytoremediation—defined as the use of plants to extract, contain, or immobilize soil contaminants—represents an innovative and economically viable alternative to conventional remediation. The main types of phytoremediation include phytoextraction, phytostabilization, rhizofiltration, phytovolatilization, and phytodegradation. However, its widespread adoption is often limited by the relatively slow growth rates of many hyperaccumulator species and the prolonged time required to significantly reduce contamination [19]. Consequently, current research prioritizes the identification and development of plant species that combine high metal accumulation capacity, substantial biomass production, and economic value [20].

A key advantage of phytoremediation is the removal of pollutants by harvesting and processing metal-laden biomass. The simplicity of biomass collection and post-harvest management—such as incineration followed by safe ash disposal or utilization of ash as a secondary raw material—enhances the practicality and sustainability of this approach [21,22,23].

Among cultivated hyperaccumulator candidates, sunflower, alfalfa, and jute have received particular attention. Sunflower (*Helianthus annuus* L.) exhibits a strong capacity for heavy metal uptake [24]. Sponge gourd (*Luffa aegyptiaca* Mill.), an annual vegetable crop, demonstrates adaptability to diverse growing conditions, though cultivar-specific differences in the uptake of cadmium (Cd), lead (Pb), and cobalt (Co), among others, have been reported [25]. Jute (*Corchorus capsularis* L.) is characterized by high biomass production and rapid growth, allowing repeated harvesting, which increases the total removal of heavy metals from the soil [26]. However, this does not necessarily reduce their concentration within plant tissues; prolonged growth and multiple harvests may, in fact, enhance cumulative metal uptake.

Soil contamination with Pb, Cd, and Co remains a critical challenge in the arid regions of Kazakhstan, where low organic matter and high carbonate content limit metal mobility and remediation efficiency. Under such constraints, sweet sorghum (*Sorghum bicolor* L.) has gained attention for its drought tolerance, high biomass productivity, and resilience to metal stress. Previous studies have shown enhanced Cd uptake with elevated BCF and, in some cases, TF > 1 in sorghum [27,28], while Pb is characteristically retained in the root zone with minimal upward transport [29]. These traits highlight *S. bicolor* as a practical candidate for phytostabilization and selective phytoextraction in dryland environments.

Taken together, recent studies [30,31,32] confirm that sorghum possesses substantial capacity for Cd and Co uptake, supported by high biomass productivity and stress tolerance. However, no studies have evaluated drought-adapted Kazakh germplasm or examined whether in vitro morphogenic performance can predict metal-uptake efficiency under arid field-relevant conditions. Therefore, the objectives of this study were to quantify genotype-dependent differences in metal accumulation and translocation, evaluate early-stage phytoextraction patterns using BCF and TF metrics, and assess the remediation potential of a pre-selected sweet sorghum genotype in arid soils of southeastern Kazakhstan. Our working hypothesis was that genotypes exhibiting superior morphogenic response in vitro would demonstrate higher shoot metal accumulation and improved phytoextraction efficiency under arid growth conditions.

The aim of this study was to evaluate the phytoremediation potential of sweet sorghum (*Sorghum bicolor* L.) under arid soil conditions in Kazakhstan by using genotypes that had previously been tested in field trials for drought tolerance and disease resistance, pre-selecting and adapting these genotypes under in vitro stress conditions, and assessing metal uptake, translocation, and early-stage accumulation patterns in the most tolerant pre-selected genotype.

We hypothesized that a sweet sorghum genotype pre-selected and adapted to arid conditions, with proven performance and disease resistance in field trials, would combine stress tolerance with root-dominated accumulation of Pb, Cd, and Co (TF < 1), making it suitable for the phytostabilization of heavy-metal-contaminated soils in the arid regions of Kazakhstan.

## 2. Results

### 2.1. Selection of Drought-Tolerant Sweet Sorghum Genotypes for Subsequent Phytoremediation Experiments

During in vitro culture of somatic cells of sweet sorghum (*Sorghum bicolor* L.), significant variation in callus induction capacity was observed among the tested genotypes (Table 1). The highest callus formation frequencies were recorded for *Hybrid-2* (69.11%), *SAB-3* (43.83%), *SABB-1* (42.31%), and *SAB-10* (40.32%). Moderate callus induction was observed in *SAB-11* (36.70%) and *Hybrid-1* (25.33%), whereas *SAB-2* exhibited an extremely low response, with only 4.47% of explants producing calls.

**Figure 1:**
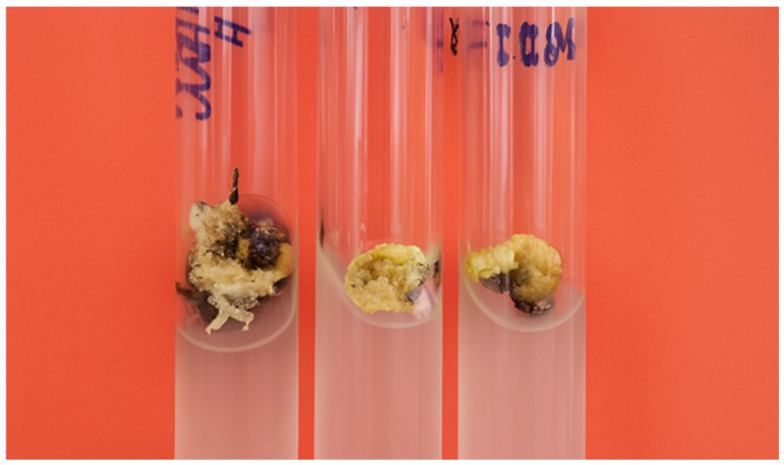
Friable, non-morphogenic calli obtained from in vitro somatic cell culture of sweet sorghum (*Sorghum bicolor* L.).

The resulting callus tissues also differed markedly in morphology and developmental potential. In most cases, the calli remained non-morphogenic, appearing as loose, undifferentiated aggregates of parenchyma-like cells (Figure 1).

Nevertheless, certain genotypes produced callus tissues exhibiting clear signs of morphogenesis, indicating their potential suitability for subsequent micropropagation and clonal propagation programs.

**Figure 2:**
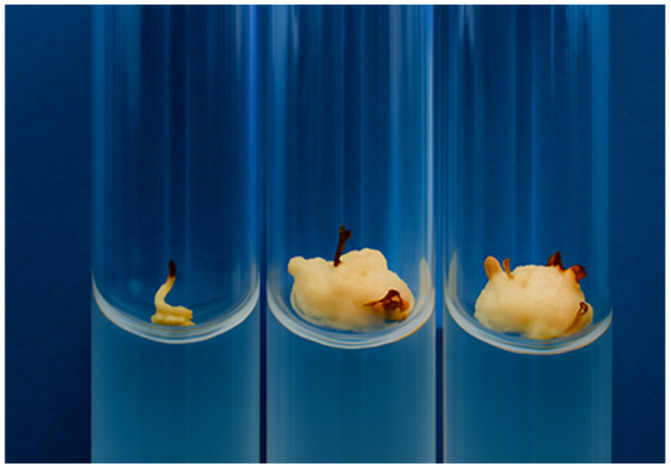
Morphogenic calli obtained from in vitro somatic cell cultures of sweet sorghum (*Sorghum bicolor* L.).

The genotypes that produced morphogenic calli with dense tissue organization and actively dividing meristematic cells were of particular interest (Figure 2).

These calli exhibited a whitish coloration and maintained a high proliferative capacity upon subculturing onto fresh nutrient media. The frequency of morphogenic callus formation varied among genotypes, with the highest values observed in *Hybrid-2* (27.94%) and *SAB-3* (23.28%) (Table 2), confirming the superior morphogenetic potential of these lines.

As a result of these studies, we assembled a collection of sweet sorghum (*Sorghum bicolor* L.) genotypes that are potentially suitable as starting material for phytoremediation-oriented breeding and further evaluation. Optimal pre-sowing seed treatments were identified, which promoted vigorous development of both the root system and aboveground tissues in seedlings. Enhanced root and leaf growth is a key physiological trait supporting overall growth intensity and, ultimately, yield potential, which is critical for both biomass production and effective phytostabilization.

The origin and breeding background of the genotypes used in this study have been described in detail in our earlier work. The *SAB* and *SABB* series represent experimental lines derived from long-term field evaluations in the arid zone of southeastern Kazakhstan. These lines were selected for their high productivity, elevated sugar content in stem juice, and resistance to major biotic stress factors, including smut, root rot, and Fusarium wilt. Field trials conducted under arid, rainfed conditions showed that plant height in these genotypes typically ranged from 197 to 277 cm, confirming their ability to produce substantial biomass under water-limited conditions. Application of silico-phosphate mineral fertilizers increased panicle mass from 0.19–0.68 g in unfertilized plants to 0.28–0.85 g in fertilized plants. Sugar content in stem juice in the best-performing genotypes reached approximately 23.0%, compared with 19.9% in the standard variety, and several experimental lines, including *SAB-4* (21.4%), *SAB-1* (20.7%), and *SAB-3* (20.3%), also exceeded the standard in sugar accumulation.

Phenological observations allowed us to classify the tested sweet sorghum genotypes into early-, mid-, and late-maturing groups, all of which demonstrated good adaptability to the arid conditions of southeastern Kazakhstan. In parallel, disease monitoring under field conditions identified genotypes with stable resistance to the major fungal and bacterial pathogens of sorghum, whereas more susceptible lines showed clear symptoms of smut, root rot, and Fusarium wilt [33,34].

Based on this combined evidence from field trials and in vitro culture, *Hybrid-2* and *SAB-3* were identified as the most promising adapted genotypes, combining high biomass productivity, sugar accumulation, disease resistance, and enhanced morphogenetic capacity in somatic cell culture. In the present phytoremediation study, we therefore selected the genotype *Hybrid-2*, a pre-adapted drought-tolerant line, for detailed analysis of Pb, Cd, and Co uptake, translocation, and phytostabilization potential in contaminated soils under controlled pot conditions.

### 2.2. Comparative Analysis of Heavy Metal Accumulation in Sorghum Tissues and Soil

**Figure 3:**
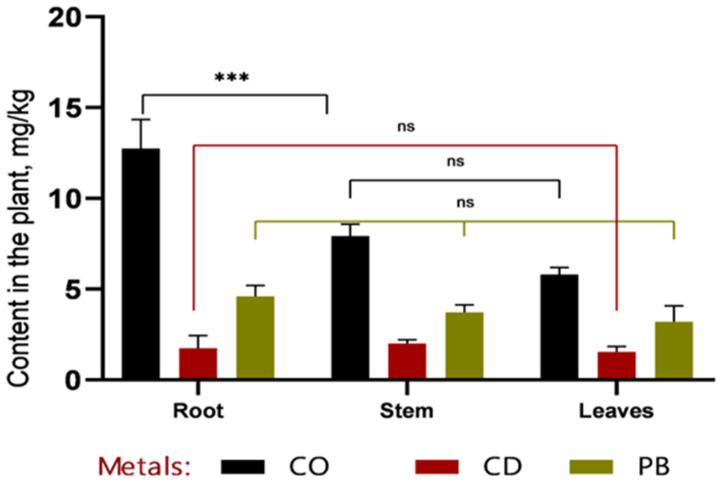
Comparative distribution of heavy metals (Co, Cd, and Pb) in roots, stems, and leaves of *Sorghum bicolor* L. after a 30-day vegetative period. The highest concentration of cobalt was observed in the roots (approximately 15 mg/kg), which was significantly higher than that in stems and leaves (*** *p* < 0.001). Cadmium and lead levels remained low and showed no statistically significant differences among plant organs (ns, *p* > 0.05). Overall, metal concentrations decreased from roots to leaves, with the most pronounced gradient observed for cobalt.

**Figure 4:**
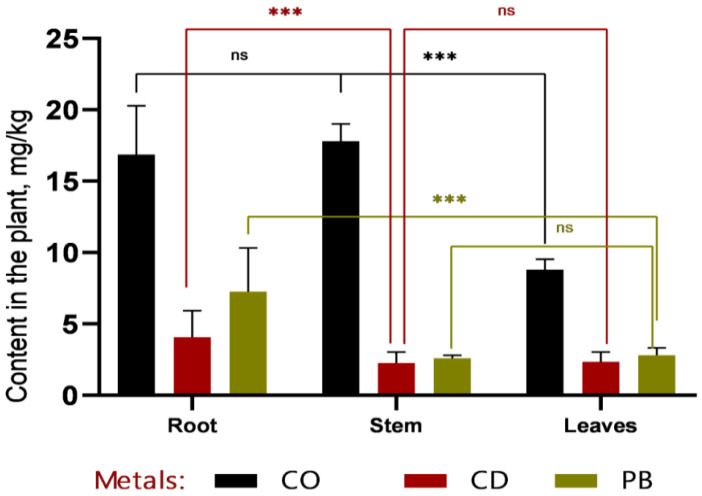
Comparative distribution of heavy metals (Co, Cd, and Pb) in plant organs after a 30-day vegetative period. Cobalt predominantly accumulated in roots and stems, with lower concentrations detected in leaves. Cadmium was present only in trace amounts, showing no marked differences among organs. Lead was primarily retained in roots and leaves, whereas its concentration in stems remained low. Data are presented as mean values ± standard errors; *** denotes *p* < 0.0001, and “ns” indicates no statistically significant difference (*p* > 0.05).

In the phytoremediation experiment employing *Sorghum bicolor* L. for the removal of heavy metals (Pb, Cd, Co) from contaminated soil, data were obtained on metal accumulation in plant tissues and concurrent changes in soil metal concentrations (Figure 3 and Figure 4).

Based on the data presented in Figure 3 and Figure 4, it can be concluded that, within the first 30 days of vegetation, heavy metals (Co, Cd, and Pb) primarily accumulated in the roots, indicating a high rate of metal uptake from soil to roots. Efficient translocation from roots to stems is particularly important for phytoremediation, as it enables the sequestration of heavy metals in easily harvestable aboveground biomass—specifically, stems. The highest cobalt concentrations were recorded in roots (12.7 ± 1.32 mg/kg at 1 MAC and 16.87 ± 2.78 mg/kg at 2 MAC), accounting for more than 50% of the total Co content in the plant. A similar pattern was observed for cadmium, with 49% of its total accumulation localized in roots, further confirming the high efficiency of metal uptake from soil. Lead accumulation in roots constituted 53% of the total Pb content in the plant.

Analysis of heavy metal concentrations 60 days after emergence revealed that, in all samples except for cobalt (Co at 1 MAC) and lead (Pb at 1 MAC), metal levels in stems remained within permissible limits.

The investigation of heavy metal accumulation dynamics across different plant organs yielded significant findings that can inform both quantitative and qualitative assessments of phytoremediation efficacy. Clear patterns of metal partitioning among roots, stems, and leaves were established, providing valuable insights for optimizing soil remediation strategies targeting specific contaminants.

**Figure 5:**
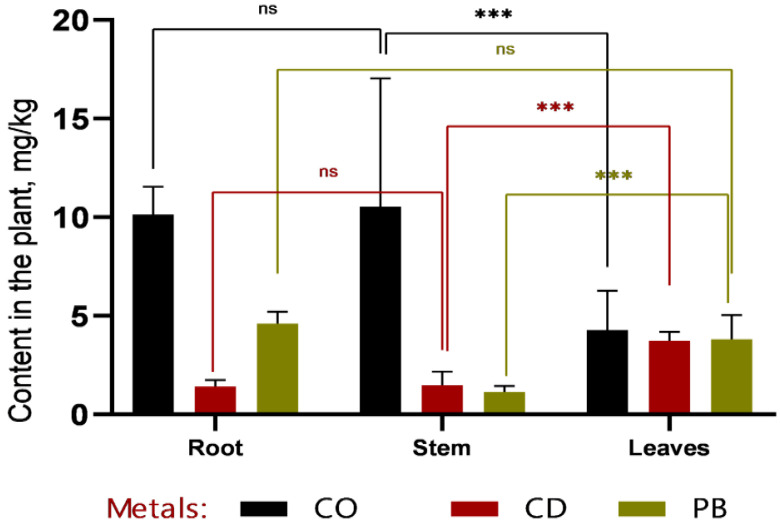
Concentrations of heavy metals (Co, Cd, and Pb) in roots, stems, and leaves of *Sorghum bicolor* L. after a 60-day vegetative period. Cobalt (Co) accumulated predominantly in roots and stems, with significantly lower levels detected in leaves. Cadmium (Cd) was present in all plant organs at low concentrations, showing a slight tendency toward higher accumulation in leaves. Lead (Pb) was primarily localized in roots and leaves, while its concentration in stems remained minimal. Asterisks denote statistically significant differences between plant organs (*** *p* < 0.001); “ns” indicates no significant difference (*p* > 0.05).

**Figure 6:**
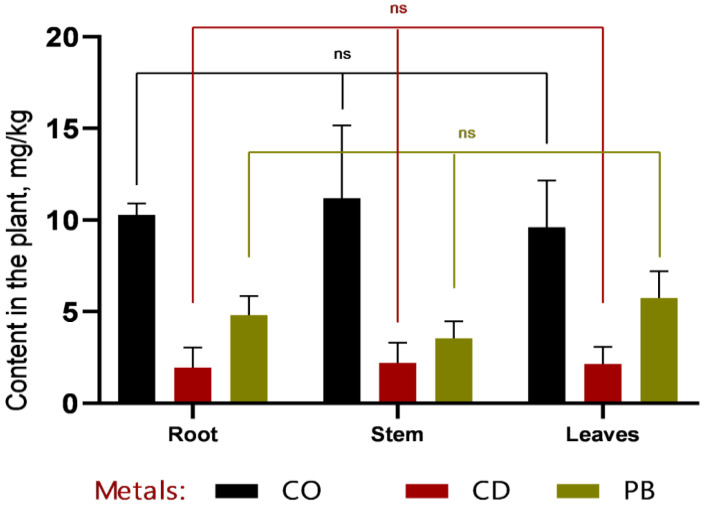
Distribution of heavy metals in sorghum organs after a 60-day vegetative period. Cobalt accumulates primarily in roots and stems, lead is predominantly found in roots and leaves, while cadmium is present in all plant organs at low concentrations. Statistically significant differences were observed only for specific metals (“ns” denotes non-significant differences, *p* > 0.05).

According to the data presented in Figure 5 and Figure 6, cobalt (Co) exhibits distinct accumulation patterns across plant tissues. At 1 MAC (maximum allowable concentration), Co is predominantly localized in leaves (8.8 mg/kg), with markedly lower concentrations in stems and roots. This suggests a high capacity of sweet sorghum to translocate and sequester cobalt in photosynthetically active tissues.

Similarly, lead (Pb) shows elevated concentrations in leaves compared to roots and stems, particularly at 2 MAC (5.7 ± 1.20 mg/kg in leaves, 4.8 ± 0.86 mg/kg in roots, and 3.5 ± 0.75 mg/kg in stems). This pattern indicates efficient root-to-shoot translocation of Pb, underscoring the potential of *Sorghum bicolor* L. for phytoextraction-based phytoremediation strategies.

In contrast, cadmium (Cd) concentrations remain consistently low across all plant organs and do not reach levels comparable to those of Co or Pb. This may reflect either lower bioavailability of Cd in the tested soil or limited physiological capacity of sorghum to absorb and translocate this metal, suggesting a more restricted role for this species in Cd-specific remediation contexts.

**Figure 7:**
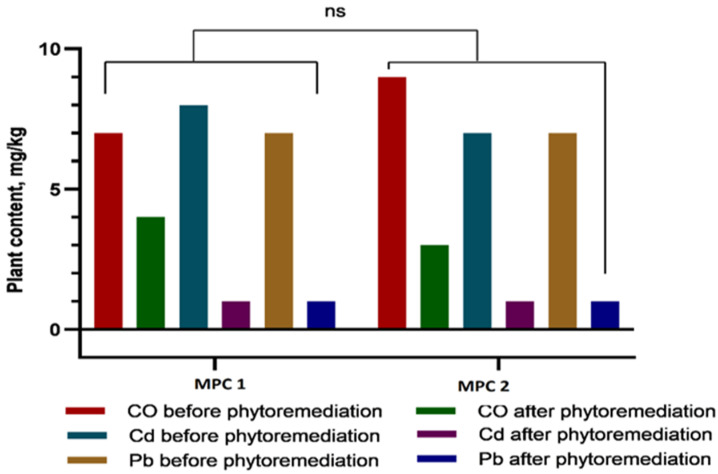
Concentrations of heavy metals (Co, Cd, and Pb) in *Sorghum bicolor* L. plants before and after phytoremediation in two experimental treatments (1 MAC and 2 MAC). Following phytoremediation, Cd and Pb concentrations decreased markedly—nearly to baseline levels—whereas Co content was reduced by approximately half. No statistically significant differences were observed between the 1 MAC and 2 MAC treatments (ns, *p* > 0.05).

Based on the changes in soil metal concentrations shown in Figure 7, the remediation efficiency (RE, %) of Hybrid-2 ranged from 30.3% to 62.7% for Co, from 81.7% to 88.1% for Cd, and from 89.3% to 89.7% for Pb at 1 MAC and 2 MAC. These values indicate a particularly high reduction in soil Cd and Pb concentrations, whereas the decrease in Co was more moderate, especially at the 1 MAC level.

### 2.3. Quantifying Metal Accumulation and Redistribution Through BCF and TF

To quantify the phytoremediation performance of sweet sorghum, the Bioconcentration Factor (BCF) and Translocation Factor (TF) were calculated from the measured metal concentrations. The values obtained for 30 and 60 days are shown in Table 3 and Table 4.

As shown in Table 3, at 30 days of growth, *Sorghum bicolor* L. predominantly accumulated absorbed metals in the root system. This is indicated by consistently higher BCF_root values compared with BCF_shoot for all examined elements, along with low TF values reflecting limited upward transfer of Co, Cd, and Pb during the early vegetative stage. These results characterize the initial phase of metal accumulation, when root-based retention plays a dominant role. To evaluate how this pattern evolves at a later developmental stage, the TF and BCF values at 60 days are presented in Table 4.

### 2.4. Correlation Analysis of Metal Accumulation Indices

To further clarify the relationships among the different accumulation indices, a Pearson correlation analysis was performed separately for the 30-day and 60-day datasets (Co, Cd, and Pb at 1 and 2 MAC; n = 6 for each time point). The results for 30 days are summarized in Table 5. Root and shoot metal concentrations were strongly and positively correlated with each other (r = 0.93, *p* < 0.01) and showed very high correlations with their respective bioconcentration factors. Root metal concentrations were highly associated with BCF_root (r = 0.96, *p* < 0.01) and BCF_shoot (r = 0.96, *p* < 0.01), whereas shoot metal concentrations were strongly correlated with BCF_shoot (r = 0.97, *p* < 0.01). In contrast, the translocation factor (TF) exhibited only weak or negligible correlations with BCF_root and BCF_shoot (r = −0.34 and r = 0.04, respectively), indicating that translocation efficiency is not directly coupled to overall accumulation intensity at this early growth stage.

The 60-day correlations (Table 6) confirmed and strengthened these patterns. Root and shoot metal concentrations were almost perfectly correlated (r = 1.00, *p* < 0.01), and both showed very strong positive associations with BCF_root and BCF_shoot (r = 0.94–0.97, *p* < 0.01). BCF_root and BCF_shoot were likewise tightly correlated (r = 1.00, *p* < 0.01), confirming that both indices reliably reflect internal metal loads at the later growth stage. In contrast, Csoil showed only weak to moderate correlations with plant-based variables (r = 0.25–0.57), and TF displayed moderate positive correlations with BCF_root and BCF_shoot (r ≈ 0.56–0.62). Taken together, these results indicate that metal accumulation in plant tissues is internally coherent at both 30 and 60 days, while translocation efficiency remains only partially coupled to total accumulation, especially at the later growth stage.

## 3. Discussion

Heavy-metal contamination represents one of the most persistent environmental challenges in Kazakhstan, particularly in arid regions where low organic matter, high carbonate content, and salinity restrict metal mobility and complicate remediation efforts. Recent assessments confirm the presence of elevated Cd, Pb, and Co concentrations in agricultural soils across Kazakhstan, together with substantial ecological risks associated with their accumulation [5,6,7,8]. Within this broader environmental context, our findings provide experimental evidence that sweet sorghum (*Sorghum bicolor* L.) can contribute meaningfully to the remediation of metal-polluted soils under arid conditions.

An important feature of our approach is that the sweet sorghum genotypes used here had already been evaluated under field conditions in the arid regions of southeastern Kazakhstan and had demonstrated agronomic stability. Field trials under rainfed conditions showed that plants reached heights of approximately 197–277 cm and produced substantial biomass even under water limitation. Application of silico-phosphate mineral fertilizers increased panicle mass from about 0.19–0.68 g in unfertilized plants to 0.28–0.85 g in fertilized plants, confirming a strong yield response to improved nutrition. Stem juice sugar content reached around 20–23% in several breeding lines, with some *SAB* genotypes exceeding the standard variety. Disease monitoring further identified genotypes with stable resistance to key sorghum pathogens, including smut, root rot, and Fusarium wilt [33,34].

Complementary in vitro experiments with somatic cell cultures revealed pronounced genotype-dependent differences in callus induction and morphogenic potential: the highest callus formation frequencies were observed in *Hybrid-2* (69.11%) and *SAB-3* (43.83%), and these same genotypes produced the largest proportion of compact, morphogenic calli (27.94% and 23.28%, respectively). Thus, the subsequent laboratory pre-selection and phytoremediation experiments were not performed on random lines, but on genotypes that were already adapted to local farming conditions and had demonstrated stress tolerance in both field and in vitro settings. This two-step strategy—field-based evaluation followed by laboratory pre-selection and pot-scale phytoremediation testing—increases the practical relevance of our findings and supports the realistic use of the selected genotype in future remediation programs.

A central outcome of our study is the clear metal-specific behavior of the pre-selected sweet sorghum genotype. Cadmium and cobalt exhibited pronounced upward mobility, with translocation factors exceeding unity at both sampling periods. This trend is consistent with previous reports demonstrating the strong Cd phytoextraction capacity of sorghum genotypes, facilitated by efficient root uptake and maintenance of shoot biomass under metal stress [27,28]. Such patterns are characteristic of an adaptive strategy in which Cd and Co are redistributed toward metabolically active tissues, enhancing the utility of sweet sorghum in phytoextraction-oriented technologies.

In contrast, lead displayed limited mobility, with substantially higher concentrations retained in the root system and BCF_root values consistently exceeding 1. The restricted translocation of Pb observed in our experiment agrees with evidence that Pb strongly binds to root tissues and cell-wall components in *S. bicolor* [29]. This mechanism creates a physiological barrier that reduces Pb movement to aerial organs and supports the application of sweet sorghum in early-stage phytostabilization. Sequestration of Pb in roots additionally limits the risk of contaminant transfer into aboveground biomass, which is important for the safe removal, disposal, or potential valorization of shoots.

The correlation analysis provided further insight into these accumulation patterns. At both 30 and 60 days, root and shoot metal concentrations were strongly and positively correlated with their respective bioconcentration factors, indicating that BCF_root and BCF_shoot reliably reflect internal metal loads. By contrast, TF showed only weak to moderate correlations with BCF_root and BCF_shoot, suggesting that translocation efficiency represents a distinct aspect of metal handling rather than a simple consequence of higher overall accumulation. This pattern is compatible with a phytoremediation strategy in which the pre-selected sorghum genotype maintains robust metal uptake while limiting excessive upward transfer, particularly in the case of Pb.

Changes in soil metal concentrations before and after plant growth provided an integrative measure of the phytoremediation effect. Based on the soil data shown in Figure 7, remediation efficiency (RE, %) of *Hybrid-2*, calculated from the decrease in soil metal concentrations, ranged from 30.3% to 62.7% for Co, from 81.7% to 88.1% for Cd, and from 89.3% to 89.7% for Pb at 1 MAC and 2 MAC. These values indicate especially strong reductions in soil Cd and Pb concentrations and a more moderate, but still substantial, decrease in Co.

Genotypic variation represents another factor influencing the remediation performance of *S. bicolor*. Previous studies have shown that biomass sorghum and energy sorghum genotypes often accumulate more Cd than sweet sorghum due to differences in root system architecture, total biomass, and metabolic allocation [33,34]. Our results are consistent with this principle: higher vegetative growth was associated with greater Cd and Co accumulation in shoots, reinforcing the close relationship between biomass production and phytoextraction efficiency.

Beyond remediation, sorghum offers additional value as a bioenergy feedstock. Although bioethanol production was not evaluated in this study, the high biomass yields observed here, together with literature demonstrating the feasibility of integrating sweet sorghum-based phytoremediation with bioethanol production, underscore the dual-purpose potential of this species [35]. Such integration can enhance the economic and environmental sustainability of remediation projects in resource-limited arid environments.

Taken together, our findings, supported by existing literature, indicate that Sorghum bicolor can function as a dual-role species: an efficient phytoextractor of Cd and Co and a reliable phytostabilizer of Pb. Its performance is shaped by the interplay of varietal traits, soil chemistry, and environmental conditions. These characteristics, combined with prior field adaptation and in vitro pre-selection under stress conditions, position sweet sorghum as a promising candidate for environmentally sustainable, large-scale remediation initiatives across the arid regions of Kazakhstan.

## 4. Materials and Methods

### 4.1. Development of a High-Productivity Genotype of Sweet Sorghum (Sorghum bicolor L.)

The study focused on sweet sorghum (*Sorghum bicolor* L.), a promising crop characterized by high biomass yield, resilience to adverse environmental conditions, and a demonstrated capacity for heavy metal accumulation—traits that render it highly valuable for phytoremediation applications.

*Sorghum bicolor* L. breeding lines and hybrids were obtained from the breeding collection of the Kazakh Research Institute of Agriculture. The panel included high-sugar lines “Kazakhstansky 20”, “AS-64”, and “Hybrid-3 line” (21.4%, 20.3%, and 20.7% sugars, respectively), along with hybrids Hybrid-1, Hybrid-2, and Hybrid-3 derived from standard regional parental combinations.

Genotypes were selected based on: adaptation to arid conditions of southeastern Kazakhstan, variation in sugar content, and preliminary differences in in vitro callus-induction response.

Immature embryos from diverse *Sorghum bicolor* L. genotypes were used as explants for in vitro somatic cell culture. The protocol for explant isolation, surface sterilization, and culture medium composition was adapted from established plant tissue culture methodologies [36,37]. Callus induction was carried out on a modified Murashige and Skoog (MS) basal medium supplemented with 2 mg/L 2,4-dichlorophenoxyacetic acid (2,4-D), 20 g/L sucrose, and 100 mg/L myoinositol, adjusted to pH 5.7. Cultures were maintained under controlled conditions at 27 ± 1 °C in the dark for 30–60 days.

The sterilization procedure followed a standard protocol widely used for cereals and sorghum tissue culture. Explants were first immersed in 70% ethanol, followed by treatment with a 10% commercial bleach solution (containing approximately 5% active sodium hypochlorite), and finally exposed to 3% hydrogen peroxide. Exposure times for each step were optimized experimentally to achieve effective surface decontamination while maintaining the viability of the explants. This combined approach is consistent with commonly reported sterilization methods for sorghum and other grasses.

Immature embryos from diverse *Sorghum bicolor* L. genotypes were used as explants for in vitro somatic cell culture. Immature embryos were collected at 10–12 days post-anthesis (DPA), corresponding to the early milk stage of seed development, when the scutellum is clearly differentiated, and embryo size reaches approximately 1.0–1.5 mm. Prior to inoculation, embryos were carefully excised under a stereomicroscope to preserve tissue integrity.

The culture medium was solidified with 0.8% agar, and explants were maintained under a 16/8 h light/dark photoperiod with a light intensity of 50–60 µmol m^−2^ s^−1^ during the regeneration phase. Subcultures were performed every 14 days to sustain tissue viability and promote consistent morphogenic development throughout the culture cycle.

The protocol for explant isolation, surface sterilization, and culture medium composition was adapted from established plant tissue culture methodologies [33,34]. Callus induction was carried out on a modified Murashige and Skoog (MS) basal medium supplemented with 2 mg/L 2,4-dichlorophenoxyacetic acid (2,4-D), 20 g/L sucrose, and 100 mg/L myoinositol, adjusted to pH 5.7. Cultures were maintained under controlled conditions at 27 ± 1 °C in the dark for 30–60 days.

To evaluate genotype productivity, a comprehensive assessment of agronomic and morphological traits was conducted at full maturity. Agronomic parameters included grain yield, seed weight, seed germination rate, and total biological productivity. Morphological characteristics encompassed plant height, panicle length and weight, and visual assessment of root system development.

The laboratory phase of genotype development was carried out at the Center for Biotechnology of Satbayev University (Kazakh National Research Technical University). Field trials and phenotypic evaluation of promising lines were conducted under the agroecological conditions of southeastern Kazakhstan at the experimental plots of the Institute of Agriculture and Crop Production. This integrated laboratory–field approach enabled a robust evaluation of the adaptive potential and abiotic stress tolerance of the selected genotypes.

### 4.2. Laboratory-Scale Model Experiment on Phytoremediation of Heavy Metal-Contaminated Soils

To assess the phytoremediation potential of sweet sorghum (*Sorghum bicolor* L.), a model experiment was conducted during the summer period of 2023 (July to September) at the Scientific Research Institute for Biological Safety Problems (Zhambyl Region, Gvardeyskiy settlement). The study was performed under semi-controlled outdoor laboratory conditions on the institute’s experimental site. Average environmental temperatures during the experimental period were approximately 30 °C (day)/24 °C (night) in July, 27 °C (day)/20 °C (night) in August, and 25 °C (day)/18 °C (night) in September, reflecting typical summer climatic conditions of the region. Plants were grown under natural sunlight without supplemental lighting.

Air-dried commercial black soil was used as the growing medium. Before use, the soil was sifted, crushed, and thoroughly homogenized to ensure a uniform structure and chemical composition. Two kilograms of prepared soil were placed in each pot. The pots were randomly arranged in blocks and rotated every five days to minimize microclimate fluctuations. All treatments were watered with distilled water of the same quality to maintain comparable soil moisture levels. These measures ensured uniformity of growing conditions and reproducibility of the experimental design.

**Figure 8:**
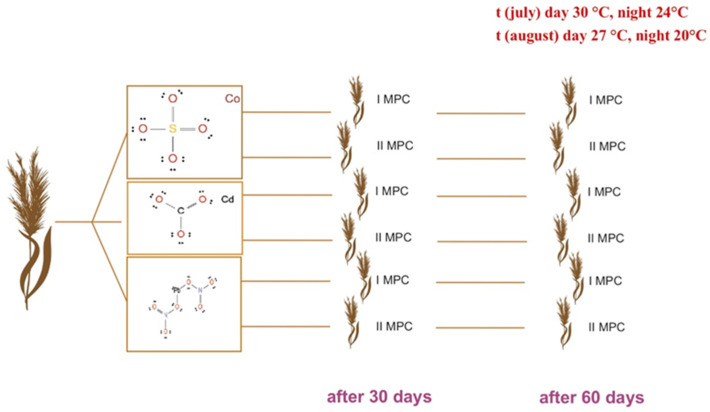
Experimental design: Sorghum plants were grown under two levels of heavy metal contamination (1× and 2× MAC) and sampled at 30 and 60 days after sowing. The experiment was conducted under natural summer conditions—in July (30 °C day/24 °C night) and August (27 °C day/20 °C night).

The experimental design included two groups: (1) a treatment group with soil artificially contaminated with heavy metal salts, and (2) a control group with uncontaminated soil. Contamination was achieved by adding aqueous solutions of lead nitrate (Pb(NO_3_)_2_), cobalt sulfate (CoSO_4_), and cadmium chloride (CdCl_2_). Each salt was dissolved in distilled water and uniformly applied to the soil to reach target contamination levels corresponding to 1× and 2× the maximum allowable concentration (MAC). The soil was thoroughly mixed to ensure a homogeneous distribution of contaminants. Control pots received identical soil and irrigation regimes but without any added pollutants (Figure 8).

Five seeds of sweet sorghum were sown per pot. All plants, in both control and treatment groups, received identical irrigation—daily watering to field capacity—to eliminate the confounding effects of water stress on growth and development.

Soil and plant sampling were carried out at two key vegetative stages: on day 30 and day 60. Harvested plants were separated into morphological components—roots, stems, and leaves. Soil samples were gently cleaned to remove adhering particles, air-dried at room temperature to a constant weight, and stored in parchment envelopes until chemical analysis. In parallel, plant explants were processed under aseptic conditions: following each sterilization step, they were rinsed 3–5 times with sterile distilled water (approximately 10–15 mL per rinse) to ensure complete removal of residual disinfectants and to prevent re-contamination prior to culture initiation.

Concentrations of heavy metals (Pb, Cd, Co) in both soil and plant tissues were determined at the laboratory of LLP “Kazakh Scientific Research Institute of Soil Science and Agrochemistry named after U.U. Uspanov.”

The uncontaminated control group enabled an objective comparison of the effects of heavy metal exposure on the growth and development of sweet sorghum under otherwise identical cultivation conditions.

### 4.3. Determination of Heavy Metal Content in Soil and Plant Tissues

In our experiment, we use MAC exactly as defined by Kazakhstan’s national soil safety standards [38]. These MAC values apply to the total metal content in soil. For the three metals tested in our study, the regulatory limits are: Cd—1 mg/kg, Co—5 mg/kg, and Pb—32 mg/kg. All experimental concentrations were prepared based on these official thresholds.

Concentrations of lead (Pb), cadmium (Cd), and cobalt (Co) were determined using atomic absorption spectrometry (AAS) on a Shimadzu AA-6200 (Shimadzu, Kyoto, Japan) instrument. Soil digestion was carried out according to ISO 11466:1995 [39], in which air-dried and homogenized soil samples are treated with a 3:1 (*v*/*v*) mixture of concentrated HNO_3_ and HCl, heated to complete dissolution of the mineral matrix, and subsequently filtered and diluted to the prescribed final volume. Plant material (roots, stems, leaves) was processed following ISO 11047:1998 [40]: after drying and grinding, samples were subjected to wet digestion using concentrated HNO_3_ with the gradual addition of H_2_O_2_ to ensure full oxidation of organic matter, followed by filtration and dilution with distilled water.

Measurements were performed in an air–acetylene flame at the following wavelengths: Pb—283.3 nm; Cd—228.8 nm; and Co—240.7 nm.

Instrument performance was monitored through routine calibration using certified standard solutions provided with the instrument. Measurements were accepted only when the calibration curve satisfied the built-in linearity criteria of the Shimadzu AA-7000 system, and the absorbance signal was stable. Selected samples were analyzed in duplicate to confirm the reproducibility of the measurements.

Remediation efficiency (RE, %) for each metal and contamination level was calculated from the change in soil metal concentrations before and after the phytoremediation experiment according to the equation:

RE (%) = [(C_initial − C_final)/C_initial] × 100

where C_initial and C_final are the soil metal concentrations (mg kg^−1^) measured before and after phytoremediation, respectively. The values of C_initial and C_final used in this calculation correspond to the soil concentration data presented in Figure 7.

### 4.4. Statistical Data Analysis

Statistical analyses were conducted using GraphPad Prism 8.0 (GraphPad Software, San Diego, CA, USA). All data are presented as mean ± standard deviation (SD). Each treatment included three biological replicates (n = 3), and each sample was analyzed in triplicate (technical replicates). Data were tested for normality using the Shapiro–Wilk test and for homogeneity of variances using Levene’s test. One-way ANOVA was applied to evaluate differences among treatments, followed by Tukey’s HSD post hoc test. Statistical significance was considered at *p* < 0.05

## 5. Conclusions

The results of this study confirm that sweet sorghum (*Sorghum bicolor* L.) can effectively contribute to the remediation of soils contaminated with lead, cadmium, and cobalt under arid conditions. A two-step approach was implemented: first, in vitro and field-based evaluation identified Hybrid-2 and SAB-3 as the most promising genotypes in terms of morphogenetic response, biomass production, and adaptation to the arid regions of southeastern Kazakhstan; subsequently, the pre-adapted genotype Hybrid-2 was selected for detailed phytoremediation testing.

In controlled laboratory pot experiments with artificially contaminated soil, atomic absorption spectrometry showed that the primary accumulation of Pb, Cd, and Co occurred in the root system, with BCF_root consistently exceeding BCF_shoot and translocation factors remaining below 1 at both sampling periods. This pattern indicates limited upward movement of metals, particularly Pb, and supports the role of sweet sorghum as an efficient phytostabilizer for this element. At the same time, overall metal uptake was sufficient to substantially reduce soil metal concentrations: remediation efficiency (RE, %) of Hybrid-2, calculated from the decrease in soil metal levels, reached approximately 82–88% for Cd and 89–90% for Pb, with a moderate but meaningful reduction (around 30–63%) observed for Co.

Collectively, these findings support our initial hypothesis that a pre-selected, drought-tolerant sweet sorghum genotype can maintain growth, produce substantial biomass, and effectively accumulate heavy metals under arid conditions. Hybrid-2, in particular, emerges as a dual-role genotype—acting as a phytoextractor of Cd and Co and a phytostabilizer of Pb—and, therefore, represents a promising candidate for environmentally sustainable remediation strategies in the metal-contaminated, arid soils of Kazakhstan. Further field-based assessments will be essential to validate these pot-scale results under real farming conditions and to optimize agronomic practices for large-scale application.

## Figures and Tables

**Table 1:** Callus induction in in vitro somatic cell cultures of sweet sorghum (*Sorghum bicolor* L.).

Genotype	Number of Isolated Embryos	Number of Calli Formed	Callus Induction Rate (%)
*SABB-1*	52	22	42.31
*SAB-2*	67	3	4.47
*SAB-3*	73	32	43.83
*SAB-10*	62	25	40.32
*SAB-11*	79	29	36.70
*Hybrid-1*	75	19	25.33
*Hybrid-2*	68	47	69.11

**Table 2:** Frequency of morphogenic callus formation in in vitro somatic cell cultures of sweet sorghum (*Sorghum bicolor* L.).

Genotype	Number of Isolated Embryos	Number of Calli Formed	Callus Induction Rate (%)
*SABB-1*	52	9	2.99
*SAB-2*	67	0	0
*SAB-3*	73	17	23.28
*SAB-10*	62	8	12.90
*SAB-11*	79	6	7.59
*Hybrid-1*	75	12	16.0
*Hybrid-2*	68	19	27.94

**Table 3:** Translocation Factor (TF) and Bioconcentration Factor (BCF) of *Sorghum bicolor* L. at 30 Days.

Metal	Soil Level	Csoil (mg·kg^−1^)	Root (mg·kg^−1^)	Shoot (mg·kg^−1^)	BCF_Root	BCF_Shoot	TF
Co	1 MAC	7.03	12.733	6.867	1.811	0.976	0.539
Co	2 MAC	9.93	16.200	13.300	1.631	1.339	0.821
Cd	1 MAC	8.43	1.733	1.767	0.206	0.210	1.020
Cd	2 MAC	7.63	4.067	2.300	0.533	0.302	0.566
Pb	1 MAC	7.50	4.600	3.467	0.613	0.462	0.753
Pb	2 MAC	7.76	7.267	2.700	0.937	0.348	0.372

**Table 4:** Translocation Factor (TF) and Bioconcentration Factor (BCF) of *Sorghum bicolor* L. at 60 Days.

Metal	Soil Level	Csoil (mg·kg^−1^)	Root (mg·kg^−1^)	Shoot (mg·kg^−1^)	BCF_Root	BCF_Shoot	TF
Co	1 MAC	7.03	10.133	9.667	1.442	1.374	0.954
Co	2 MAC	9.93	10.267	10.393	1.034	1.047	1.012
Cd	1 MAC	8.43	1.400	1.300	0.166	0.154	0.929
Cd	2 MAC	7.63	2.600	2.167	0.341	0.284	0.833
Pb	1 MAC	7.50	4.267	3.767	0.569	0.502	0.883
Pb	2 MAC	7.76	4.800	4.633	0.619	0.597	0.965

**Table 5:** Pearson correlation coefficients (r) between metal accumulation indices in sweet sorghum (*Sorghum bicolor* L.) at 30 days of growth (Co, Cd, and Pb at 1 and 2 MAC; n = 6).

Variable 1	Variable 2	r
Root metal concentration	Shoot metal concentration	0.93 **
Root metal concentration	BCF_root	0.96 **
Root metal concentration	BCF_shoot	0.96 **
Shoot metal concentration	BCF_root	0.80
Shoot metal concentration	BCF_shoot	0.97 **
BCF_root	BCF_shoot	0.90 *
Csoil (total metal in soil)	Shoot metal concentration	0.67
Csoil (total metal in soil)	Root metal concentration	0.42
Csoil (total metal in soil)	TF	0.53
TF	BCF_root	−0.34
TF	BCF_shoot	0.04

Note. Values represent Pearson correlation coefficients (r) calculated for the 30-day dataset (Co, Cd, and Pb at 1 and 2 MAC; n = 6). Given the small sample size, the correlations are interpreted as exploratory. Based on critical r values for df = 4, |r| ≥ 0.81 is considered significant at *p* < 0.05 (*) and |r| ≥ 0.92 at *p* < 0.01 (**).

**Table 6:** Pearson correlation coefficients (r) between metal accumulation indices in sweet sorghum (*Sorghum bicolor* L.) at 60 days of growth (Co, Cd, and Pb at 1 and 2 MAC; n = 6).

Variable 1	Variable 2	r
Root metal concentration	Shoot metal concentration	1.00 **
Root metal concentration	BCF_root	0.96 **
Root metal concentration	BCF_shoot	0.97 **
Shoot metal concentration	BCF_root	0.94 **
Shoot metal concentration	BCF_shoot	0.96 **
BCF_root	BCF_shoot	1.00 **
Csoil (total metal in soil)	Shoot metal concentration	0.30
Csoil (total metal in soil)	Root metal concentration	0.25
Csoil (total metal in soil)	TF	0.57
TF	BCF_root	0.56
TF	BCF_shoot	0.62

Note. Values represent Pearson correlation coefficients (r) calculated for the 60-day dataset (Co, Cd, and Pb at 1 and 2 MAC; n = 6). Given the small sample size, the correlations are interpreted as exploratory. Based on critical r values for df = 4, |r| ≥ 0.92 is considered significant at *p* < 0.01 (**).

## Data Availability

Data is contained within the article.

## Abbreviations

The following abbreviations are used in this manuscript:

AAS	Atomic absorption spectroscopy
MPC	Maximum permissible concentration
As	Arsenic
Cd	Cadmium
Cr	Chromium
Hg	Mercury
Cu	Copper
Pb	Lead
Ni	Nickel
Zn	Zinc
MAC	Maximum allowable concentration
MS	Murashige and Skoog
BCF	Bioconcentration Factor
TF	Translocation Factor
RE	Remediation efficiency
